# Proteome-Wide Multipoint Internal Calibration Curves for Evaluating Peptide-Level Linearity in Relative Quantitative Proteomics

**DOI:** 10.1021/acs.jproteome.5c00179

**Published:** 2025-12-23

**Authors:** Cristina Chiva, Zahra Elhamraoui, Julia Morales-Sanfrutos, Olga Pastor, Eduard Sabidó

**Affiliations:** † 16372 Centre for Genomic Regulation, The Barcelona Institute of Science and Technology, Dr Aiguader 88, 08003 Barcelona, Spain; ‡ 16770 Universitat Pompeu Fabra, Dr Aiguader 88, 08003 Barcelona, Spain

## Abstract

Mass spectrometry (MS)-based proteomics is known for its high accuracy in quantifying peptides and proteins using various calibration strategies including internal and external calibration curves. While external multipoint calibration curves are created from serial dilutions, they often fail to account for sample-specific matrix effects. In contrast, internal calibration curves account for the sample matrix but face scalability and cost challenges for whole proteome analyses. In this manuscript, we present a novel TMT-based multipoint internal calibration curve strategy, which enables the generation of internal calibration curves for all peptides identified within a proteome in a single experiment to assess their linearity prior relative quantification. We applied this strategy to human ovarian cancer cells to evaluate the linear quantitative responses of all of the identified peptides and reveal the significant proteome changes associated with cisplatin treatment.

## Graphical abstract

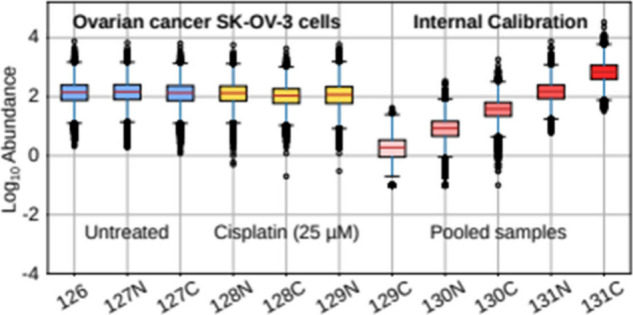

## Introduction

Mass spectrometry (MS)-based proteomics is well-known for its ability to identify and quantify thousands of peptides and proteins in complex biomedical samples.^1,2^ In proteome-wide studies, protein relative quantification is performed by comparing peptide areas to derive fold-changes between conditions. In these experiments, the linear range of quantification and the positioning of each endogenous peptide within this range are often not evaluated, which can affect the accuracy and precision of subsequent protein relative quantification. In contrast, in quantitative targeted proteomics experiments, various calibration strategies are employed, including internal and external calibration curves, to establish the relationship between peptide signal and peptide concentration.^3–6^ While these curves establish the range of linearity between instrument response and analyte concentration, they also have limitations in accounting for sample-specific matrix effects, as linearity is not directly determined within each sample. This issue is particularly significant when dealing with patient cohorts and complex samples, where variations in sample matrices can affect the linear quantitative behavior and, therefore, the accuracy in relative proteome quantification. To address these limitations, internal calibration curves have been proposed, using either matrix-matched approaches^7^ or by spiking isotopically labeled standards directly into the samples.^8–10^ These internal isotopically labeled standards are often used in the single-point internal calibration mode, in which a known concentration of the standard is added to determine the response factor, assuming a linear relationship through zero. While this method is quick and resource-efficient, it does not fully capture the complexity of the response curve. A few years ago we developed an isotopologue multipoint calibration strategy (ImCal) that uses a mixture of isotopically labeled peptides at different concentrations to establish a multipoint internal calibration curve for the peptides of interest.^11^ This method facilitated determining the linear range of quantification for each targeted analyte directly in its sample, and it ensured precise and accurate absolute quantification of specific peptides in targeted proteomics applications.^11–13^ However, the use of multiple isotopologue peptides increases the cost of each targeted assay, and its application to entire proteomes remains limited due to the challenges of scaling, making it suitable only for clinical projects in which a small number of peptides are measured across a large number of samples.

In this study, we relied on the ImCal concept to evaluate the peptide quantitative linearity in the entire proteome by developing a tandem mass tag (TMT)-based multipoint internal calibration curve strategy for all of the peptides identified within a proteome. This approach is based on TMT-labeled serial dilutions of total protein extract that are used to generate internal calibration curves together with the samples of interest within a single experiment. These multipoint internal calibration curves enable researchers to assess the quantitative linearity of each identified peptide in its context matrix prior its use in relative quantification.

## Materials and Methods

Human ovarian cancer cells (SK-OV-3 from American Type Culture Collection, ATCC) were cultured in the presence or absence of 25 μM cisplatin in triplicates. Protein extracts (600 μg) were reduced with TCEP (1.8 μmol, 37 °C, 60 min), alkylated in the dark with iodoacetamide (3.6 μmol, 25 °C, 30 min), and digested with endoproteinase LysC (1:10 w:w, 37 °C, over 6h, Wako, cat # 129–02541) and trypsin (1:10 w:w, 37 °C, over 8h, Promega cat # V5113). After digestion, the peptide mixes were acidified with formic acid and desalted with a MicroSpin C18 column (The Nest Group, Inc.). A pooled sample was generated by combining equal amounts of the three biological replicates from both conditions to create a pool from which to generate an internal calibration curve with five different serial dilutions. Individual samples were labeled using tandem mass tags (TMT-11) according to the manufacturer instructions and the experimental design specified in Table 1. This experiment was performed in triplicate. TMT mixes were fractionated using a basic pH reversed-phase fractionation. Twelve fractions were analyzed in using an Orbitrap Eclipse mass spectrometer (Thermo Fisher Scientific, San Jose, CA, USA) coupled to an EASY-nLC 1000 (Thermo Fisher Scientific (Proxeon), Odense, Denmark) with a 90 min gradient.^14^ Data acquisition was done using the real-time synchronous precursos selection MS3 acquisition method (RTS-SPS-MS3).^15^ The scan sequence began with an MS1 spectrum, and in each cycle of data-dependent acquisition analysis, following each survey scan, the most intense ions were selected for fragmentation. Fragment ion spectra were produced via collision-induced dissociation (CID) at normalized collision energy of 35% and they were acquired in the ion trap mass analyzer in “Turbo” mode. MS2 spectra were searched in real time using the algorithm embedded in the instrument control software and the canonical human database from Uniprot (version 2021). MS2 spectra with an Xcorr greater than or equal to 1 and less than 10 ppm precursor mass error triggered the submission of an MS3 spectrum to the instrument. MS3 spectra were collected using the multinotch MS3-based TMT method, in a way where ten MS2 fragment ions were captured in the MS3 precursor population using isolation waveforms with multiple frequency notches. MS3 precursors were fragmented by high energy collision-induced dissociation (HCD) at a normalized collision energy of 65% and acquired in the Orbitrap analyzer. The mass spectrometry proteomics data have been deposited to the ProteomeXchange Consortium via the PRIDE partner repository with the data set identifier PXD059628.^16^

**Table 1:** Experimental Design for samples, Tandem Mass Tags, and Protein Amounts (μg) Multiplexed in a Single TMT Batch

**TMT channel**	**Sample**	**Amount (μg)**
126	SK-OV-3 Cells Untreated Replicate 1	25
127N	SK-OV-3 Cells Untreated Replicate 2	25
127C	SK-OV-3 Cells Untreated Replicate 3	25
128N	SK-OV-3 Cells Treated (25 μM cisplatin) Replicate 1	25
128C	SK-OV-3 Cells Treated (25 μM cisplatin) Replicate 2	25
129N	SK-OV-3 Cells Treated (25 μM cisplatin) Replicate 3	25
129C	Calibration curve pool	0.25
130N	Calibration curve pool	1.25
130C	Calibration curve pool	6.25
131N	Calibration curve pool	25
131C	Calibration curve pool	125

Acquired spectra were analyzed using the Proteome Discoverer software suite (v2.4, Thermo Fisher Scientific) and the Mascot search engine (v2.6, Matrix Science).^22^ Data was searched against a customized database including the Uniprot human canonical database plus a list of common contaminants and all the corresponding decoy entries (version 2021).^23^ For peptide identification, a precursor ion mass tolerance of 7 ppm was used for the MS1 level, trypsin was chosen as enzyme, and up to three missed cleavages were allowed. The fragment ion mass tolerance was set to 0.5 Da for the MS2 spectra. Oxidation of methionine and N-terminal protein acetylation were used as variable modifications, whereas carbamidomethylation on cysteines, TMT6plex in Lysines and in peptide N-terminal were set as a fixed modification. False discovery rate (FDR) in peptide identification was set to a maximum of 5%. The list of identified peptides was filtered to remove those peptides without quantitation values, i.e. those labeled as “NoQuanLabels”, “NoQuanValues” and “ExcludedByMethod” by the Proteome Discoverer software suite were excluded from the subsequent quantification analysis. Peptides were quantified using the reporter ion intensities in MS3. Reporter ion intensities were adjusted to correct for the isotopic impurities of the different TMT reagents according to the manufacturer specifications. A linear regression was fit for peptides with at least three valid quantitative values within the calibration curve. Statistical inference was performed MSstatsTMT (v4.16).^19–21^

This research did not involve human or animal participants, and according to the CRG, all experiments were performed with proper ethics.

## Results

To evaluate the quantitative linearity of each identified peptide within a proteome, human ovarian cancer cells (SK-OV-3) were cultured in triplicate with and without cisplatin (25 μM), and the resulting proteomes were digested with endoproteinase LysC and trypsin. Simultaneously, a pooled sample was prepared to generate serial dilutions for the internal multipoint calibration curve (Figure 1A,B and Table 1). Tandem mass tags (TMT-11) were employed to label all of the samples and serial dilutions, which were combined to form a single multiplexed TMT experiment. After basic pH reversed-phase fractionation, the TMT samples were analyzed using a 90 min gradient on an Orbitrap Eclipse mass spectrometer, and an acquisition method with real-time search and synchronous precursor selection MS3 analysis (RTS-SPS-MS3).^14,15^ The acquired data were processed using Proteome Discoverer (v2.4) and all the mass spectrometry proteomics data have been deposited to the ProteomeXchange Consortium via the PRIDE partner repository^16^ with the data set identifier PXD059628.

**Figure 1:**
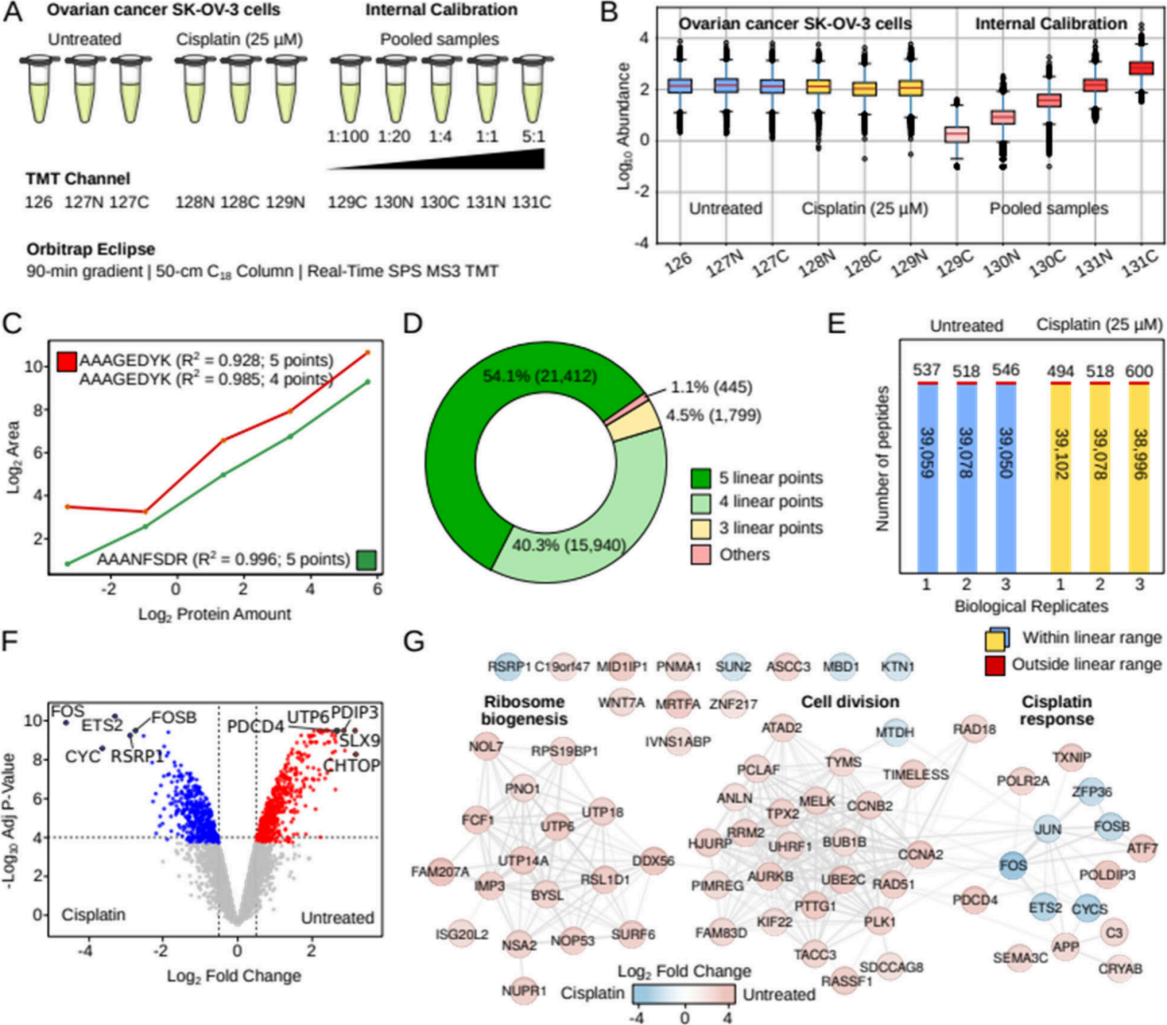
A) Human ovarian cancer cells (SK-OV-3) were cultured in triplicate with and without cisplatin treatment (25 μM) and subsequently digested with trypsin. An internal multipoint calibration curve was generated using a pooled sample with six different dilutions ranging from 5:1 to 1:100. B) Experimental log-transformed peptide abundance values (MS3 reported ion intensities) corresponding to the three biological replicates of human ovarian cancer cells (SK-OV-3) cultured with and without cisplatin and to the serial dilutions of the internal calibration curve. C) Linear regression fit for peptides [K].AAANFSDR.[S] (green) with 5 linear points (R^2^ > 0.99), and [K].AAAGEDYK.[A] (red) with four linear points (R^2^ > 0.98). D) Donut chart illustrating the classification of peptide linearity based on the count of linear points with R^2^ > 0.95. A linear fit was applied to all valid quantitative points for each peptide, and the R^2^ value was calculated. If R^2^ was below 0.95, the lowest concentration value was iteratively removed until R^2^ exceeded 0.95. The remaining quantitative values represent the number of linear points for that peptide. E) Number of endogenous peptides from the ovarian cancer cell lines that lay within the linear range established with the internal multipoint calibration curves. F) Volcano plot with proteins with significant abundance changes in human ovarian cancer cells (SK-OV-3) with and without cisplatin treatment (25 μM) using linear peptides. G) Functional protein–protein interaction networks (String-db) with the proteins exhibiting the most significant fold-changes in protein abundance in cisplatin treated human ovarian cancer cells (SK-OV-3) using linear peptides.

Using this TMT-based approach, we first evaluated the linear behavior of the TMT-labeled internal calibration curves for 39,596 peptides with at least three valid quantitation values—out of a total of 46,641 identified peptides (). Based on the log-transformed MS3 signals obtained from the TMT-labeled calibration curves, we calculated the number of linear points for each peptide. Briefly, a linear fit was applied to all valid quantitative points for each peptide, and R^2^ was calculated. If R^2^ was below 0.95, the lowest concentration value was iteratively removed until R^2^ exceeded 0.95. The remaining quantitative values represent the number of linear points for that peptide. With this approach, we observed that the majority of identified peptides exhibited an excellent linear quantitative response within the serial dilutions tested, with approximately 95% of them demonstrating complete or nearly complete linear regression curves, i.e. four or five linear points with R^2^ > 0.95 (Figure 1C,D). Not only were most calibration curves complete, but when missing values did occur, these missing values were predominantly found at the lowest values of the calibration curves (i.e., 89.7% of the missing points were found in the lowest concentration), thus aligning with the expectations for the data. The excellent linear quantitative response observed in this study contrasts with previous quantitative results reported at the MS1 and MS2 levels,^7,17,18^ and it is likely attributable to the enhanced interference removal achieved through the synchronous precursor selection (SPS) MS3 quantification, as well as the fact that potential coisolated interferences will rarely contribute to the corresponding TMT channel.

After evaluating the linear behavior of the TMT-labeled dilution curves for all identified peptides, we aimed to assess the reproducibility of this quantitative behavior across different batches. To achieve this, we conducted two additional TMT-labeled experiments, following the same experimental design, sample preparation, data acquisition, and data analysis protocols as those previously described. The analysis of the three TMT-calibrated replicate experiments resulted in the identification and quantification of 57,951 peptides, with approximately 70% (39,224 peptides) being identified and quantified in at least two out of the three batches (). This result highlights a common challenge in analyzing multiple TMT batches, where slight variations in the list of identified peptides reduce the completeness across batches. Nevertheless, for the peptides identified in all batches, we evaluated whether their quantitative behavior remained consistent. We observed that approximately 90% (35,077) of the peptides exhibiting complete or nearly complete linear regression dilution curves,—i.e. four or five linear points with R^2^ > 0.95 in one batch, also demonstrated a complete or nearly complete linear quantitative behavior in the other batches in which they were identified. Some peptides exhibited four linear quantitative points in one batch, while showing five in another, and vice versa. In the batches where only four linear quantitative points are observed, the peptides consistently display lower intensity in the MS3 spectra compared to the same peptide in the other batches. This observation could be attributed to the precise timing of the sampling events for fragmentation during chromatographic elution, to small differences in R^2^ values around 0.95, and to specific matrix effects from coeluting peptides present in each of the three batches ().

Finally, we applied the TMT calibration curves to a model system of ovarian cancer (SK-OV-3 cell line) treated with and without cisplatin (25 μM) to quantify the proteome remodeling that occurs after treatment. First, we assessed whether the endogenous peptides fell within the linear range of quantification established by the internal multipoint calibration curves constructed for each peptide. We observed that the vast majority of peptides identified in human ovarian cancer cells were within the linear range of quantification in the different replicates assessed (Figure 1E). This observation enables accurate and precise relative quantification for most proteins either through direct comparison of the MS3 reported ion intensities, or by adjusting these intensities using the individualized regression lines derived from the calibration curves available for each peptide. As a direct consequence of this finding, the fold changes in protein abundances obtained by directly comparing reporter ion signals were similar to those derived from calibrated reporter ion signals (). However, for some proteins, discrepancies arise in the estimated fold-changes and p-values depending on whether quantification is performed using all endogenous peptides or only those within the linear range of quantitation, leading to differences in the set of significantly changing proteins (Table 2 and ). This highlights that the decision to include all peptides or restrict the analysis to those with linear quantitative behavior can impact the overall statistical significance of certain proteins and, therefore, influence the biological conclusions drawn from an experiment. Following these observations, we conducted the proteome relative quantification of the ovarian cancer model with and without cisplatin treatment using only endogenous peptides within the linear range of quantification with MSstats (v4.12.1).^19–21^ This relative quantification allowed us to discern the significant changes in the proteome of the ovarian cancer cell lines following cisplatin treatment, which primarily affected proteins associated with the ribosome biogenesis, cell cycle, nucleic acid metabolic processes and, as expected, the cellular response to cisplatin (Figure 1F,G, ).

**Table 2:** Examples of Discrepancies in Protein Relative Quantification between Treated and Untreated (Cisplatin 25 μM) SK-OV-3 Ovarian Cancer Cells When Using All Identified Peptides or Only Those with a Linear Quantitative Behavior

Example 1: Protein P42575 (Caspase-2; CASP2_HUMAN)			
**Peptide**	**Calibration Curve**	**R** ^**2**^	**Notes**
[R].ELIQAK.[V]	Nonlinear	-	*Discarded: Less than 3 linear points*
[K].LQNFAQLPAHR.[V]	4 Linear Points	R^2^_4_ = 0.975	
	**Log2FC**	**Adj. Pvalue**	
Using all peptides	0.48	0.110	
Using only linear peptides	0.85	0.001	

Example 2: Protein P20585 (DNA mismatch repair protein Msh3; MSH3_HUMAN)			
**Peptide**	**Calibration Curve**	**R** ^**2**^	**Notes**
[R].ATSVSVQDDR.[I]	4 Linear Points	R^2^_4_ = 0.996	
[R].FHSPFIVENYR.[H]	4 Linear Points	R^2^_4_ = 0.999	
[K].LDDAVNVDEIMTDTSTSYLLCISENK.[E]	4 Linear Points	R^2^_4_ = 0.987	*Discarded: Abundance in “Untreated” sample below calibration curve*
[R].VQTESLQER.[F]	5 Linear Points	R^2^_5_ = 0.993	
	**Log2FC**	**Adj. Pvalue**	
Using all peptides	–0.49	0.017	
Using only linear peptides	–0.33	0.168	

Example 3: Protein Q15434 (RNA-binding motif, single-stranded-interacting protein 2; RBMS2_HUMAN)			
**Peptide**	**Calibration Curve**	**R** ^**2**^	**Notes**
[R].GLQPGTTDQDLVK.[L]	Nonlinear	-	*Discarded: Less than 3 linear points*
[K].GYGFVDFDSPSAAQK.[A]	5 Linear Points	R^2^_5_ = 0.979	
[K].TPPGVPAPSDPLLCK.[F]	5 Linear Points	R^2^_5_ = 0.994	
	**Log2FC**	**Adj. Pvalue**	
Using all peptides	0.62	0.017	
Using only linear peptides	0.46	0.156	

Example 4: Protein Q9UL54 (Serine/threonine-protein kinase TAO2; TAOK2_HUMAN)			
**Peptide**	**Calibration Curve**	**R** ^**2**^	**Notes**
[R].AGFGAEAEKLAR.[R]	5 Linear Points	R^2^_5_ = 0.977	*Discarded: Abundance in “Treated” sample above calibration curve*
[R].QIQEHEQDSALR.[E]	4 Linear Points	R^2^_4_ = 0.998	
	**Log2FC**	**Adj. Pvalue**	
Using all peptides	–1.67	0.003	
Using only linear peptides	–0.10	0.652	

Beyond the use case just highlighted, using the TMT-based strategy to assess peptide-level linearity in relative quantification could also enhance results in more sensitivity-challenging scenarios, such as low-input and single-cell proteomics, where a greater number of peptides are likely to fall within the noise and thus be outside the linearity range. In these scenarios, the increased proportion of nonlinear peptides could impact relative quantification if they are not identified and filtered out beforehand, thereby enhancing the value of the TMT-based strategy presented here. To illustrate the effect of using nonlinear peptides in scenarios that challenge sensitivity, we took advantage of the existing dilutions within the calibration curve to calculate their expected protein ratios to the 1:1 dilution. This analysis shows that employing only the peptides previously designated as linear results in significantly reduced dispersion around the expected theoretical protein ratios, and that the effect is especially pronounced when calculating ratios involving lower dilutions, where quantitative linearity is often compromised (). Finally, to further demonstrate the value of internal multipoint calibration curves, we simulated more challenging scenarios by adding increasing background noise to the ovarian cancer experimental data set. This was accomplished by adding constant background offsets of 20, 50, or 200 units to the measured values of each TMT channel (). Higher background noise increased the number of nonlinear peptides () and amplified the discrepancies in the calculation of protein fold-changes when using all identified peptides versus using only linear peptides (). Moreover, having an internal calibration curve not only helped identify peptides within the linear range of quantification but also enabled the calibration of endogenous peptide intensities, robustly recovering the original fold-changes despite higher background noise ().

## Conclusion

In conclusion, we developed a tandem mass tag (TMT)-based multipoint internal calibration curve strategy that extends the benefits of internal calibration to assess the quantitative linearity range for each identified peptide in a proteome before its use in relative quantification. By applying this approach to human ovarian cancer cells with real-time synchronous precursor selection (SPS) MS3 quantification, we demonstrated that the majority of identified peptides exhibit a linear quantitative response within the serial dilutions tested and that most endogenous peptides fall within the linear range of quantification. Our experimental findings, together with the observations in simulated data sets with higher background noise, highlight the importance of this TMT-based approach as a method to assess peptide-level linearity and to identify peptides outside the linear quantification range before performing relative protein quantification, while also providing insights into proteome remodeling in ovarian cancer cells in response to cisplatin treatment.

## Supplementary Material











## Data Availability

The mass spectrometry proteomics data described in this manuscript is available at the ProteomeXchange Consortium via the PRIDE partner repository with the data set identifier PXD059628.

## Notes

R code for MSstats: R code for the statistical assessment of the proteomics data performed in MSstats. Supplementary Figure S1: Number peptide sequences identified in each batch and their overlap. Supplementary Figure S2: Examples of linear response for different peptides across batches. Supplementary Figure S3: Differences between the logaritmic fold changes obtained with the raw MS3 intensities and those obtained with calibrated MS3 intensities using the TMT calibration curve. Supplementary Figure S4: Enrichment plots of significant proteins in treated and untreated (cisplatin 25 μM) SK-OV-3 ovarian cancer cells. Supplementary Figure S5: Estimated protein fold-change (logarithmic scale) across different dilutions (1:100, 1:20, 1:4 and 5:1) from the calibration curve compared to the 1:1 dilution using all or only linear peptides wit 5 linear points. Supplementary Figure S6: Classification of peptide linearity and fold change correlation between treated and untreated (cisplatin 25 μM) SK-OV-3 ovarian cancer in the ovarian cancer experimental data set with artificially added background noise. (PDF)
Supplementary Table S1: List of proteins and peptides identified and quantified in the TMT-based experiments. (XLSX)
Supplementary Table S2: MSstats relative quantification output with fold-changes and adjusted p-values comparing proteins abundance between untreated and cisplatin-treated (25 μM) SK-OV-3 ovarian cancer cells. (XLSX)
Supplementary Table S3: List of peptides identified and quantified in the TMT-based experiments with simulated increasing background noise. (XLSX)
Supplementary Table S4: MSstats relative quantification output with fold-changes and adjusted p-values comparing proteins abundance between untreated and cisplatin-treated (25 μM) SK-OV-3 ovarian cancer cells with higher background noise. (XLSX)
