# Supplementary material for: Proteome-Wide Multipoint Internal Calibration Curves for Evaluating Peptide-Level Linearity in Relative Quantitative Proteomics
Source: J Proteome Res. 2025 Dec 23;25(2):1204–10. doi: 10.1021/acs.jproteome.5c00179 (PMC12887998; doi:10.1021/acs.jproteome.5c00179)
Supplement: Supplementary file 1 [file pr5c00179_si_001.pdf]

Supporting Information

Proteome-wide multipoint internal calibration curves for evaluating peptide-level linearity in relative quantitative proteomics

Cristina Chiva[1,2,#], Zahra Elhamraoui[1,2,#], Julia Morales-Sanfrutos[1,2], Olga Pastor[1,2], Eduard Sabidó[1,2,*]

1. Centre for Genomic Regulation, Dr Aiguader 88, 08003 Barcelona, Spain

2. Universitat Pompeu Fabra, Dr Aiguader 88, 08003 Barcelona, Spain

# Equal contribution

* Corresponding Author

Table of Contents

- **R code for MSstats:** R code for the statistical assessment of the proteomics data performed in MSstats.
- **Supplementary Figure S1:** Number peptide sequences identified in each batch and their overlap.
- **Supplementary Figure S2:** Examples of linear response for different peptides across batches.
- **Supplementary Figure S3:** Differences between the logaritmic fold changes obtained with the raw MS3 intensities and those obtained with calibrated MS3 intensities using the TMT calibration curve.
- **Supplementary Figure S4:** Enrichment plots of significant proteins in treated and untreated (cisplatin 25 μM) SK-OV-3 ovarian cancer cells.
- **Supplementary Figure S5:** Estimated protein fold-change (logarithmic scale) across different dilutions (1:100, 1:20, 1:4 and 5:1) from the calibration curve compared to the 1:1 dilution using all or only linear peptides wit 5 linear points.
- **Supplementary Figure S6:** Classification of peptide linearity and fold change correlation between treated and untreated (cisplatin 25 μM) SK-OV-3 ovarian cancer in the ovarian cancer experimental dataset with artificially added background noise.
- **Supplementary Table S1:** List of proteins and peptides identified and quantified in the TMT-based experiments.
- **Supplementary Table S2:** MSstats relative quantification output with fold-changes and adjusted p-values comparing proteins abundance between untreated and cisplatin-treated (25 μM) SK-OV-3 ovarian cancer cells.
- **Supplementary Table S3:** List of peptides identified and quantified in the TMT-based experiments with simulated increasing background noise.
- **Supplementary Table S4:** MSstats relative quantification output with fold-changes and adjusted p-values comparing proteins abundance between untreated and cisplatin-treated (25 μM) SK-OV-3 ovarian cancer cells with higher background noise.

R code for MSstats

```
# Load Packages
library(MSstats)
library(MSstatsTMT)
library(MSstatsPTM)
# Package versions
# MSstats version 4.17.1
# MSstatsTMT version 2.17.0
# MSstatsPTM version 2.11.4

# Read data
data = read.delim("insert your quantification dataset filepath",sep=      )
                  annot_file = read.csv("insert your annotation filepath")
data = PDtoMSstatsTMTFormat(input = data,
                              annotation = annot_file,
                              which.proteinid ='Proteins',
                                                      use_log_file = FALSE)
data = unique(as.data.frame(data))

# use MSstats for protein summarization
summarized = MSstatsTMT::proteinSummarization(data,
               method = 'msstats',
               global_norm = TRUE,
               reference_norm = TRUE,
               remove_norm_channel  = TRUE,
               remove_empty_channel = TRUE,
               MBimpute = FALSE,
               maxQuantileforCensored = 0.999)

# use to create data summarization plots
dataProcessPlotsTMT(summarized,
                      type= "Enter ProfilePlot or QCPlot Here",
                      ylimUp = FALSE,
                      ylimDown = FALSE,
                      which.Protein = "Enter Protein to Plot Here",
                      originalPlot = TRUE,
                      summaryPlot =FALSE,
                      address = FALSE)

# Create the contrast matrix
contrast.matrix = NULL
comparison = matrix(c(1, -1),nrow=1)
contrast.matrix = rbind(contrast.matrix, comparison)
row.names(contrast.matrix)=c("Cond-A vs Cond-B")
colnames(contrast.matrix)=c("Cond-A","Cond-B")

# Model-based comparison
model = MSstatsTMT::groupComparisonTMT(summarized,
               contrast.matrix = contrast.matrix,
               moderated = TRUE,
               adj.method = "BH",
               remove_norm_channel = TRUE,
               remove_empty_channel = TRUE
               )
groupComparisonPlots(data=model$ComparisonResult,
                      type="Enter VolcanoPlot, Heatmap, or ComparisonPlot",
                      which.Comparison="all",
                      which.Protein="all",isPlotly=FALSE,
                      address="")
```

Supplementary Figure S1

Venn diagram with the number peptide sequences (with modifications) identified in each batch and their overlap.

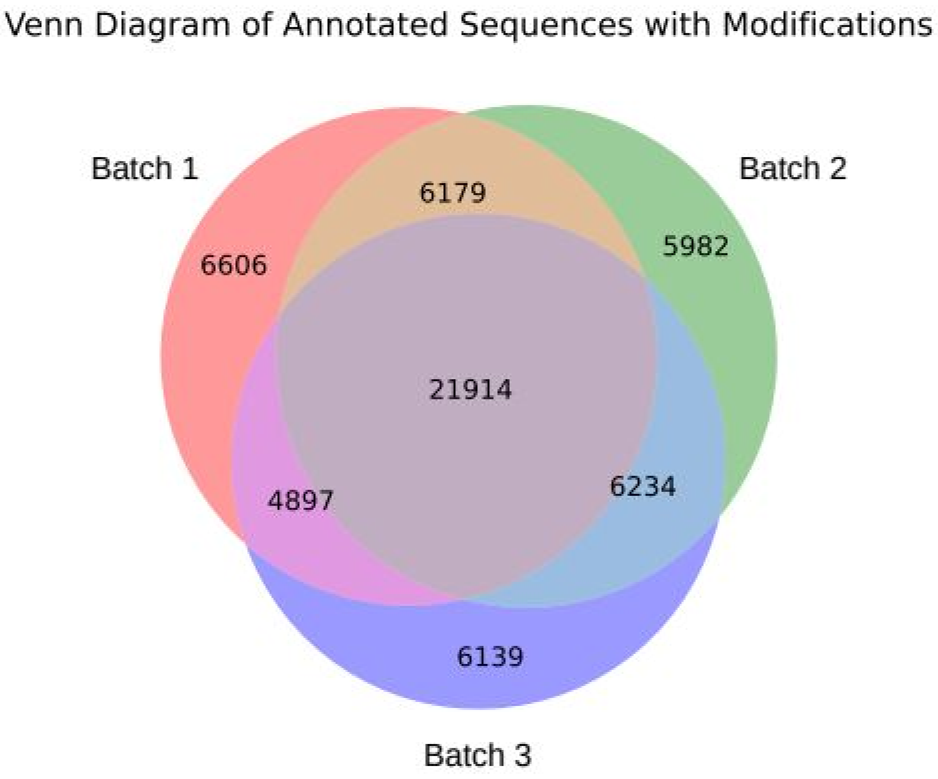

Supplementary Figure S2

A) Example of the different linear response of peptide [K].TESASVQGR.[N] across batches. The peptide shows five linear quantitative points in two different batches and only four in the last batch. B) Extracted ion chromatograms for the peptide [K].TESASVQGR.[N] across the three batches, demonstrating that the fragmentation event occurred at similar times in the first two batches, while in the third batch it was triggered earlier, well before the chromatographic apex. This timing difference may account for the variation in the number of linear quantitative points observed among the batches. C) Boxplots comparing the log-transformed MS3 signal corresponding to the highest concentration in the calibration curves for peptides exhibiting variability in the number of linear quantitative points across batches. The data indicate that, regardless of the precise underlying cause, the signal is consistently lower in the batch where the peptide displays fewer linear quantitative points.

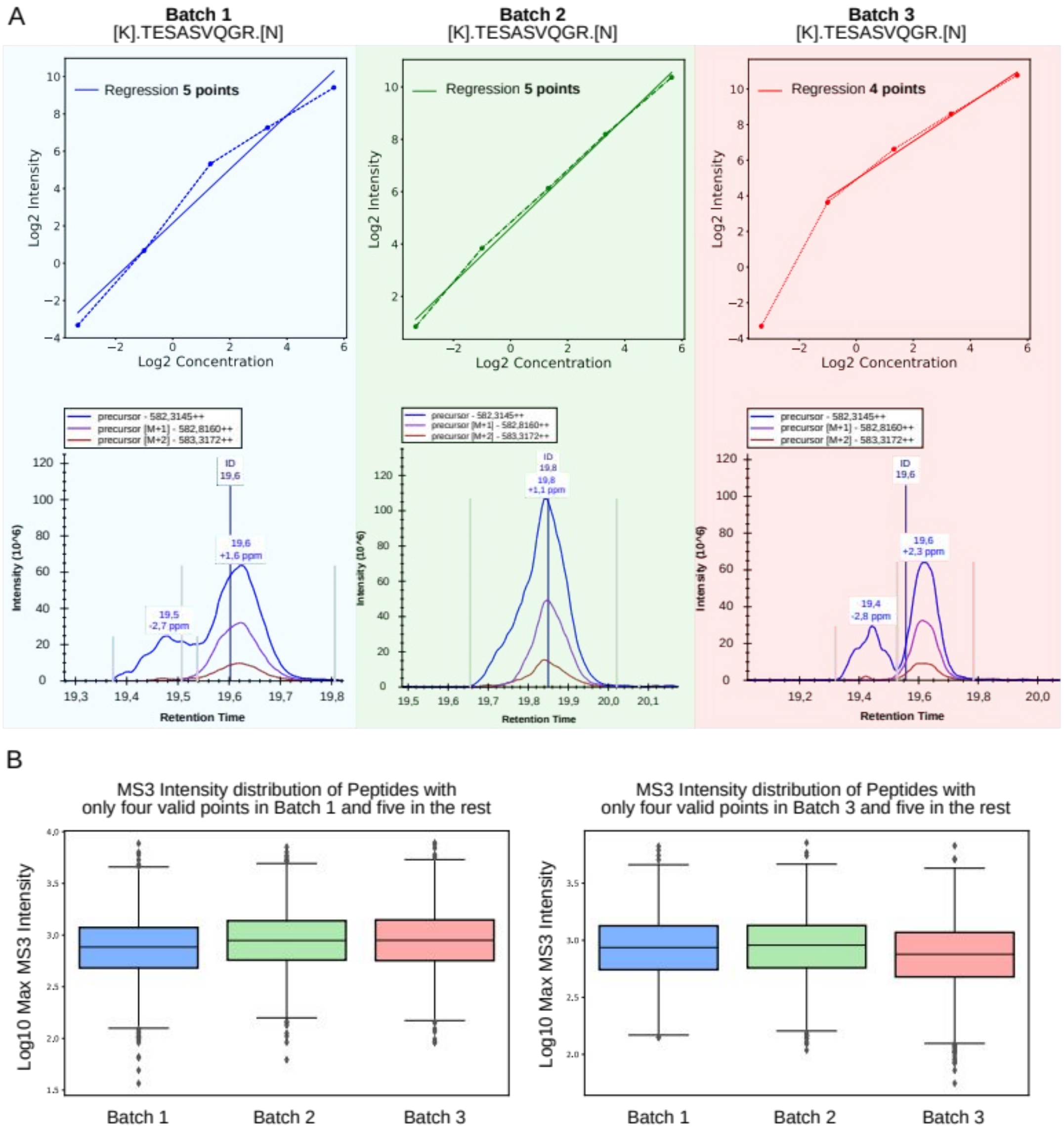

Supplementary Figure S3

Distribution of the difference (delta) between the logaritmic fold changes obtained with the raw MS3 intensities and those obtained with calibrated MS3 intensities using the TMT calibration curve.

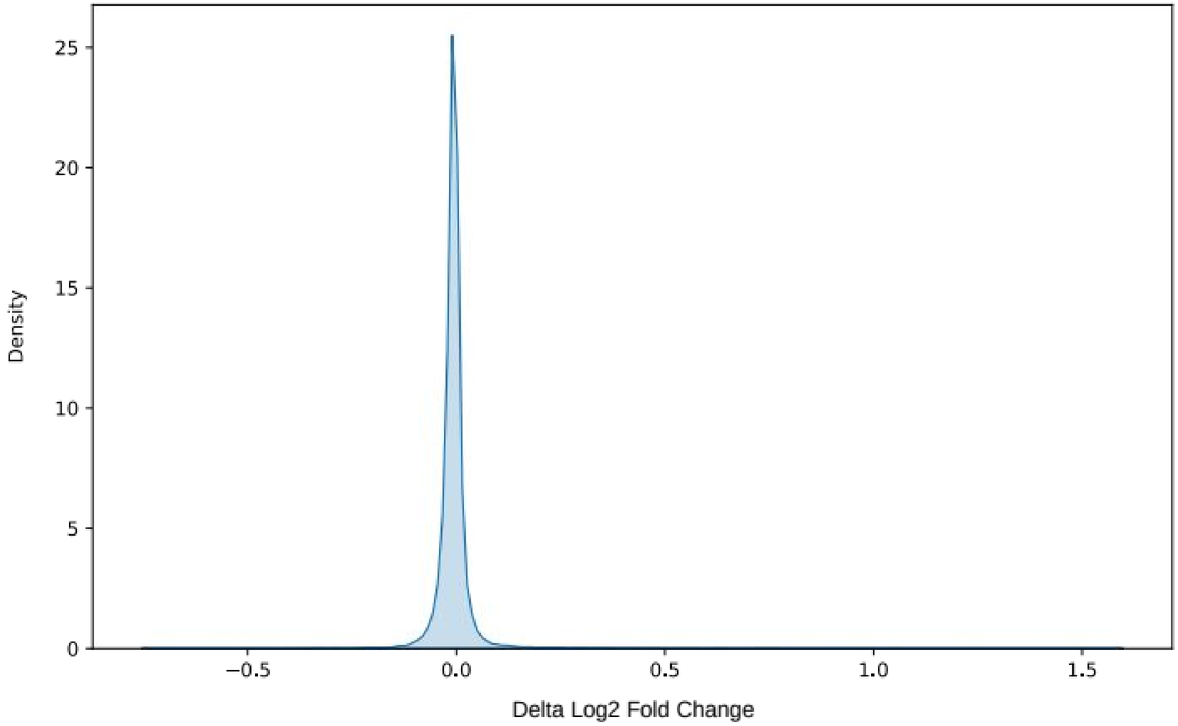

Supplementary Figure S4

Enrichment plots obtained with the proteins showing a significant change in abundance between untreated and cisplatin-treated (25 μM) SK-OV-3 ovarian cancer cells.

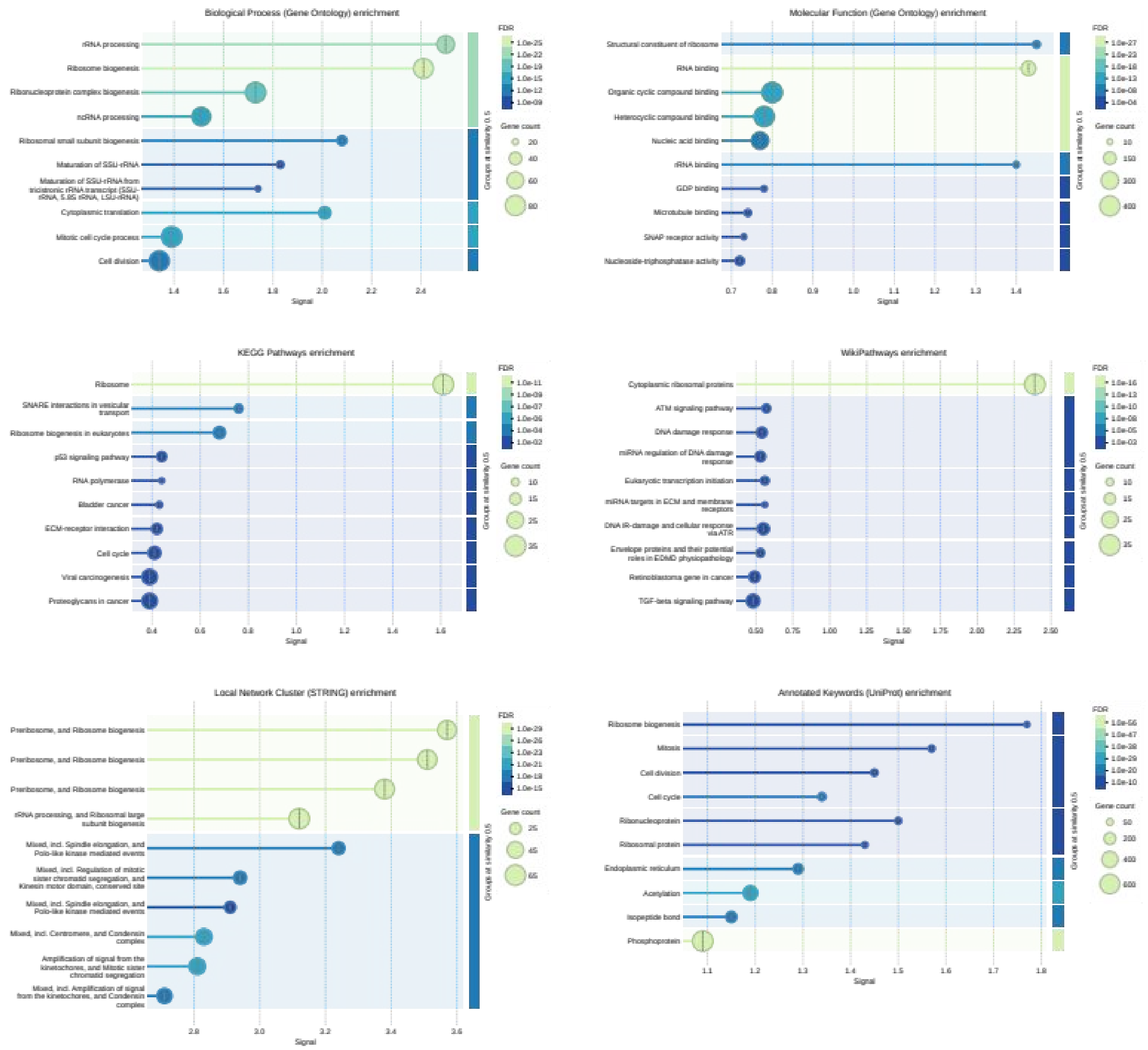

Supplementary Figure S5

Estimated protein fold-change (logarithmic scale) across different dilutions (1:100, 1:20, 1:4 and 5:1) from the calibration curve compared to the 1:1 dilution using all or only linear peptides wit 5 linear points.

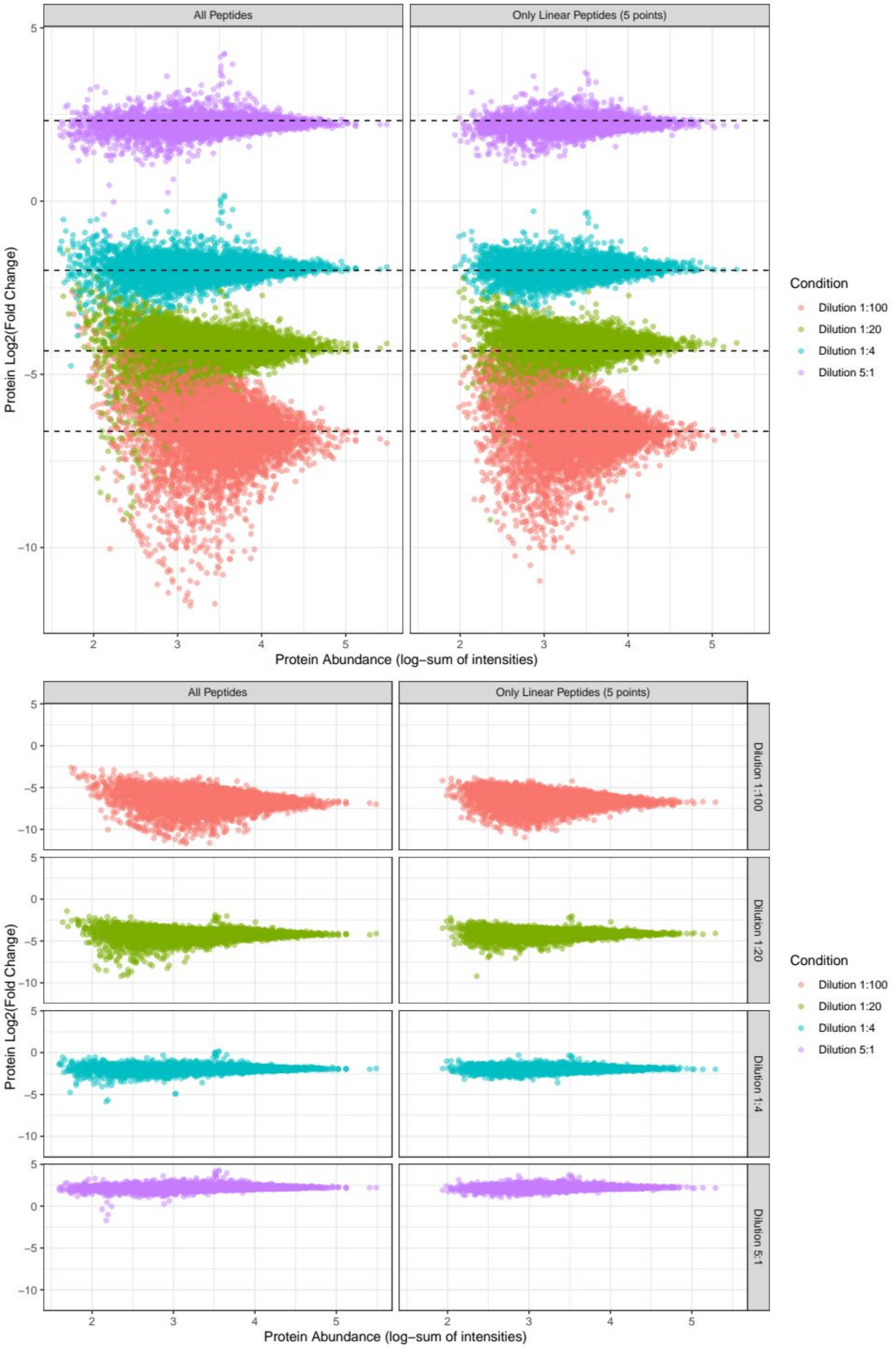

Supplementary Figure S6

A) Donut chart illustrating the classification of peptide linearity based on the count of linear points with $R^2 > 0.95$ in the ovarian cancer experimental dataset with artificially added background noise (0 units, 20 units, 50 units and 200 units of background noise). A linear fit was applied to all valid quantitative points for each peptide, and R^2 was calculated. If R^2 was below 0.95, the lowest concentration value was iteratively removed until R^2 exceeded 0.95. The remaining quantitative values represent the number of linear points for that peptide. B) Comparison of the estimated fold change (log-scale; treated and untreated SK-OV-3 cells; cisplatin 25 μM) obtained using either all identified peptides or only linear peptides within the same level of background noise. C and D) Comparison of the original fold change (log-scale; treated and untreated SK-OV-3 cells; cisplatin 25 μM) with the estimated fold change at different levels of background noise (50 and 200 units) using either raw intensities from all identified peptides, or the calibrated intensities from only the linear peptides.

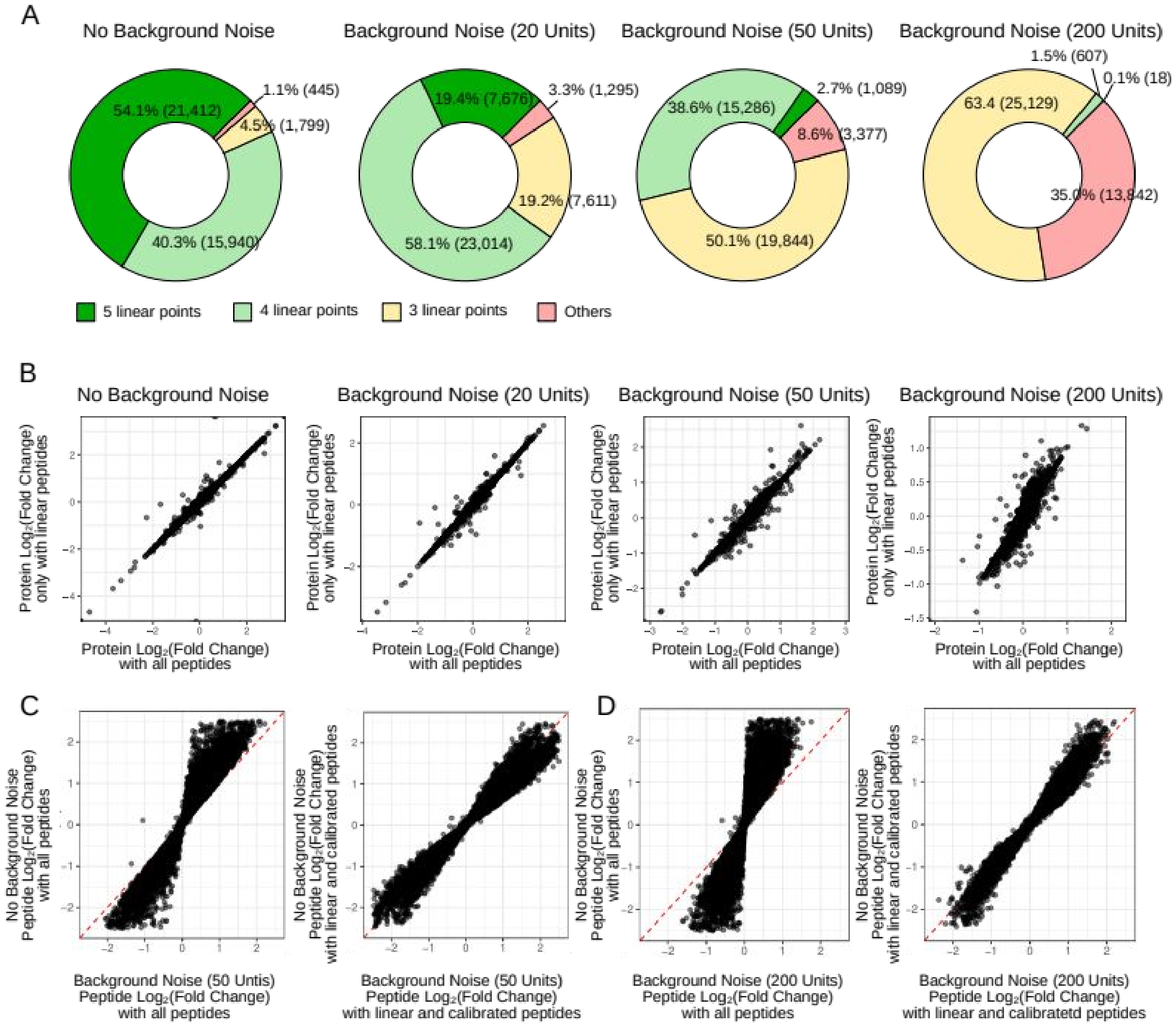

Supplementary Table S1

List of proteins and peptides identified and quantified in the TMT-based experiments using Proteome Discoverer (v2.4).

Supplementary Table S2

MSstats relative quantification output with fold-changes and adjusted p-values comparing proteins abundance between untreated and cisplatin-treated (25 μM) SK-OV-3 ovarian cancer cells.

Supplementary Table S3

List of peptides identified and quantified in the TMT-based experiments with simulated increasing backgroud noise.

Supplementary Table S4

MSstats relative quantification output with fold-changes and adjusted p-values comparing proteins abundance between untreated and cisplatin-treated (25 μM) SK-OV-3 ovarian cancer cells with higher background noise.
